# Pore-Discriminative Pervaporation of Xylene Isomers Through In Situ Synthesized MIL-100(In) Membranes

**DOI:** 10.3390/membranes15090261

**Published:** 2025-08-29

**Authors:** Jinsuo Yu, Chenyang Jiang, Yanjun Wang, Zemin Li, Yawei Gu, Rujing Hou, Yichang Pan

**Affiliations:** State Key Laboratory of Materials-Oriented Chemical Engineering, College of Chemical Engineering, Nanjing Tech University, Nanjing 210009, China; 202361204294@njtech.edu.cn (J.Y.); 202121104045@njtech.edu.cn (C.J.); wyjhgd@njtech.edu.cn (Y.W.); lzm@njtech.edu.cn (Z.L.); 202362042052@njtech.edu.cn (Y.G.); panyc@njtech.edu.cn (Y.P.)

**Keywords:** membrane separation, metal–organic framework, pervaporation, xylene isomers

## Abstract

Efficient xylene isomers’ separation remains a challenge due to their similar kinetic diameter and boiling points, particularly for the separation of the immediate size of meta-xylene (MX). A metal–organic framework (MOF) membrane offers the opportunity to realize the isomers’ separation due to the highly tunable pore size and pore environment. Herein, an In-based hierarchic MOF (MIL-100) with a size of 0.77 nm was screened, aiming at the realization for isomer separation through pore size matching. Meanwhile, the polar microenvironment in the MOF channel built through trimesic acid ligands contributes to the higher affinity to the MX relative to the PX. With the equimolar feed mixture of MX/PX, the optimal membrane demonstrated a total flux of 7.6 kg·m^−2^·h^−1^ and an MX/PX separation factor of 2.54 at room temperature through pervaporation. Such performance highly indicates the possibility for efficient liquid xylene separation in future.

## 1. Introduction

Xylene isomers, primarily comprising para-xylene (p-xylene), meta-xylene (m-xylene), and ortho-xylene (o-xylene), are predominantly obtained through the catalytic reforming process of crude oil. P-xylene serves as a key raw material for producing polymers like polyethylene terephthalate (PET) and polybutylene terephthalate (PBT), while m-xylene and o-xylene are primarily used in manufacturing isophthalic acid and phthalic anhydride, respectively [1]. The nearly identical molecular sizes (PX: 0.67 nm, MX: 0.71 nm, OX: 0.74 nm) and close boiling points (PX: 138.4 °C, MX: 139.2 °C, OX: 144.4 °C) of xylene isomers render them extremely challenging to separate. Currently, industrial-scale separation of xylene isomers primarily relies on three conventional techniques: distillation, crystallization, and adsorption. Each method presents significant limitations: (1) distillation requires over 300 theoretical plates, leading to prohibitively high energy costs [1]; (2) crystallization demands cryogenic conditions (~220 K operation temperature) while yielding unsatisfactory efficiency (single-pass PX recovery rate of merely 60–65%) [2]; (3) adsorption separation offers superior energy efficiency and separation performance for xylene isomer purification relative to the aforementioned methodologies. The most commonly used materials in this field are zeolites, carbon materials, polymers, and silica gel. A representative example is the Parex units, which utilize simulated moving bed (SMB) technology to recover para-xylene [3]. However, these conventional methods face two fundamental limitations: (1) they rely on a single separation mechanism (typically size exclusion or equilibrium adsorption), and (2) they exhibit high energy intensity during regeneration. Due to these complexities, xylene isomer separation is recognized as one of the “Seven chemical separations to change the world” [2].

In this context, membrane-based pervaporation separation has emerged as an energy-efficient alternative, attracting significant attention due to its potential to overcome these limitations through the simultaneous combination of solution-diffusion and molecular sieving mechanisms. Several membrane types have been explored for this application, including (1) MFI-type zeolite membranes [4,5,6], which suffer from high manufacturing costs and processing difficulties; (2) polymeric membranes [7,8], which exhibit limited separation selectivity; and (3) mixed-matrix membranes (MMMs) [9,10,11,12,13,14], which face challenges including interfacial defects and uneven filler distribution. Each membrane type presents distinct limitations that hinder their industrial implementation. Metal–organic frameworks (MOFs) have garnered particular attention for isomer separation due to their uniform pore size distribution and highly tunable pore microenvironments. For instance, several studies have demonstrated the effectiveness of MOFs in selective adsorption and membrane-based separation. Notably, Huang et al. developed polydopamine-modified MIL-160 membranes that achieved exceptional xylene isomer separation performance, with a high flux of 467 g·m^−2^·h^−1^ and a separation factor of 38.5 for p-/o-xylene mixtures at 75 °C due to the high adsorption and diffusion selectivity of PX in MIL-160 [15]. Uemiya et al. prepared UiO-66 membranes on alumina supports with in situ solvothermal ligand conditioning technology, which exhibited a flux of 370 g·m^−2^·h^−1^ and a separation factor of 1.2 for p-/m-xylene at an equimolar feed due to the high mobility of p-xylene in the pore space of the UiO-66 [16]. Schröder et al. synthesized four iso-structural MFM-300, demonstrating a molecular discrimination mechanism in MFM-300 for xylene isomers via refining the pore size. This material achieved xylene isomer separation factors of 2.9 (MX/PX) and 2.1 (OX/PX) due to combined molecular sieving and m-xylene preferential adsorption [17].

However, a critical limitation of current MOF-based separations lies in their predominant application to vapor-phase systems, where a high separation factor is often achieved at the expense of low feed partial pressures and limited throughput [18,19,20]. This poses a significant challenge for industrial-scale implementation, where high-capacity liquid-phase separation is essential. The urgency of advancing liquid-phase MOF membrane separations was underscored by Lively et al., who demonstrated the enrichment of p-xylene from 50 to 81 mol% in a liquid p-/o-xylene mixture using a carbon molecular sieve membrane [21]. Thus, the development and screening of MOF membranes tailored for liquid xylene isomer separation represent a crucial step toward bridging the gap between laboratory-scale achievements and industrial applications, further propelling membrane technology in the field of isomer separation.

The separation of m-xylene, which possesses intermediate molecular dimensions among xylene isomers, presents a formidable challenge for conventional size-sieving separation methods. This challenge was recently addressed by Xing et al., who achieved breakthrough performance using MIL-160(Al), demonstrating an exceptional m-xylene uptake capacity of 1.3 mmol/g coupled with an impressive m-/p-xylene separation factor of 5.6 [22]. To this end, we selected MIL-100(In), a framework with a three-dimensional mesh topology due to its abundant oxygen sites, which arise from the coordination between indium metal centers and benzene-1,3,5-tricarboxylate (BTC) linkers, enhancing m-xylene affinity. Additionally, its 0.77 nm pore size allows xylene isomers to enter and diffuse through the channels, ensuring high permeation flux. As anticipated, the membrane demonstrated exceptional performance in liquid-phase xylene separation via pervaporation at room temperature, achieving a flux of 7.6 kg·m^−2^·h^−1^ and a separation factor of 2.54 for m-xylene/p-xylene. These results highlight the potential of pore microenvironment engineering in MOF membranes for isomer separation. This work not only provides a viable strategy for liquid-phase xylene separation but also accelerates the development of advanced membrane materials for industrial applications.

## 2. Experimental Section

### 2.1. Materials

Indium chloride (InCl_3_, 99.9%) was purchased from Shanghai Macklin Biochemical Co., Ltd., Shanghai, China. 1,3,5-benzenetricarboxylic acid (H_3_BTC, 98%) was obtained from Shanghai Aladdin Biochemical Technology Co., Ltd., Shanghai, China. N, N-dimethylformamide (DMF, 98%) and ethanol (C_2_H_6_O, 99.9%) were purchased from Wuxi Yasheng Chemical Reagent Co., Ltd., Wuxi, China. Deionized water and α-Al_2_O_3_ substrates were homemade in a laboratory, α-Al_2_O_3_ (CR6) powder was purchased from Baikowski, Shanghai, China. All purchased materials were used as received without further purification.

### 2.2. Preparation of MIL-100 Particles and Membranes

For MIL-100(In) membrane fabrication, a porous α-Al_2_O_3_ disk was used as support (2 mm in thickness, 22 mm in diameter, 120 nm in average pore diameter, 40% porosity). Cross-sectional SEM images marked the support and membrane layer. According to the previous work [23] with minor modification, MIL-100(In) membranes were fabricated by the solvothermal method. To optimize the membrane quality, several key parameters were systematically investigated, including (1) the metal-to-ligand molar ratio (varied from 1:0.5 to 1:6), (2) precursor concentration (ranging from 10 to 90 mmol/L), (3) solvent composition (with DMF adjusted between 0~20 mL and ethanol between 0~17.5 mL), (4) and synthesis time (6~48 h) and temperature (90~180 °C). Taking the optimal membrane recipe as an example, 1,3,5-benzenetricarboxylic acid (0.588 g) was added into N, N-dimethylformamide (17.5 mL) and sonicated for 10 min until completely dissolved, followed by the addition of ethanol (17.5 mL) with stirring for another 10 min at 25 °C to obtain a homogeneous ligand solution. Indium chloride (0.619 g) was added in deionized water (5 mL) and sonicated for 5 min until completely dissolved into a transparent liquid. This metal solution was slowly poured into the ligand solution and stirred for 10 min in order to mix well. Finally, the above synthesized liquid was transferred and sealed in a stainless steel high-pressure kettle containing a PTFE liner. The kettle was placed in an oven at 120 °C for 24 h. After the reaction and cool down, the membrane was taken out and carefully rinsed with DMF and ethanol followed by soaking in DMF and ethanol, respectively, for 12 h to remove excess reactants. For the particles, the white precipitate formed in the PTFE liner along with the membrane synthesis was washed by fresh DMF and ethanol three times, respectively, and then collected by centrifugation followed by drying in an oven for 12 h at 60 °C.

### 2.3. Pervaporation Test

**Figure 1:**
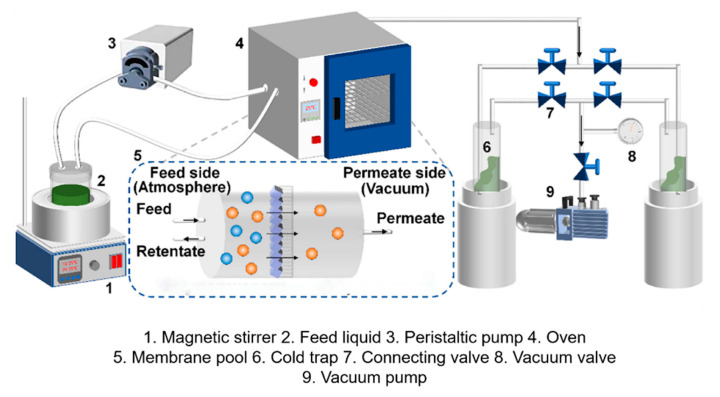
Diagram of pervaporation setup applied in this work [24].

The xylene isomer feed solution was prepared by mixing PX and MX in a 1:1 mass ratio. The membrane with an effective area of 3.8 cm^2^ was placed in a stainless steel cell sealed with a rubber o-ring gasket made of perfluoropolyether material that is resistant to high temperatures and an organic solvent. Before the pervaporation test, the membrane should be stabilized with the raw material solution for 12 h to remove residual activating solvents in the pores to ensure the accuracy of the test. As shown in Figure 1, the feedstock liquid was connected to the homemade pervaporation device via a peristaltic pump. Upon turning on the peristaltic pump, the feedstock liquid can pump into the upper side of the membrane module and circulate around the feed side at a flow rate set at 60 mL·min^−1^. For the permeate side, a glass cold trap that has been placed in liquid nitrogen was connected to the vacuum pump which was controlled at 0 MPa to provide the driving force between the membrane. The collection trap was pre-weighed to *M*1 (kg) and then put into liquid nitrogen, continuously cooled and stabilized to −196 °C using the liquid nitrogen at the periphery of the cold trap, and the condensed xylene isomer through the membrane was collected at the cold trap. The entire process of pervaporation testing was maintained for 24 h. At the end of the test, the frost on the outside of the cold trap was wiped off with a towel, it was weighed again as *M*2 (kg), and the xylene isomer fractions on the feedstock and permeate sides were analyzed for their compositions using a liquid chromatograph (GC-9790 PLUS). The total permeation flux *J* (kg·m^−2^·h^−1^) was defined as follows:

$$J=\frac{M2-M1}{∆t\cdot A}$$

where *M*2 − *M*1 is the total weight of the permeate (kg), *A* is the effective membrane area (m^2^), and ∆*t* is the collection time (h).

The separation factor (*α*_MX/PX_) was calculated as follows:

$$\alpha_{\mathrm{MX}/\mathrm{PX}}=\frac{Y_{\mathrm{MX}}/Y_{\mathrm{PX}}}{X_{\mathrm{MX}}/X_{\mathrm{PX}}}$$

where *Y*_PX_ and *Y*_MX_ are the mass fractions of PX and MX in the permeate side, and *X*_PX_ and *X*_MX_ are the mass fractions of PX and MX in the feedstock side.

### 2.4. Characterizations

An X-ray diffractometer (XRD, Rigaku Smartlab TM 9 kW powder diffractometer, Tokyo, Japan, 40 kV, 40 mA) equipped with a Cu Kα radiation source (λ = 1.54 Å) was used to characterize the crystal structure of the MIL-100 samples. Scanning electron microscopy (SEM, S4800, Hitachi, Tokyo, Japan) was used to observe the morphology of MIL-100 particles and membrane samples. A Fourier transform infrared spectrometer (FT-IR, Nicolet IS 10, Waltham, MA, USA) was used in order to detect the changes in the characteristic groups and chemical bonds during the synthesis of the MIL-100 samples. A thermogravimetric analyzer (TGA, Netzsch STA 449, Selby, Germany) was used to examine the thermal stability of the MIL-100 samples under a nitrogen atmosphere with a flow rate of 50 mL·min^−1^. Based on the adsorption–desorption data, the specific surface area and pore size of the MIL-100 samples were determined by multipoint BET (Brunauer–Emmett–Teller, BSD-660M A3M, Beijing, China) and HK (Horvath–Kawazoe, Beijing, China) methods.

## 3. Results and Discussion

### 3.1. Characterization of MIL-100(In) Powder

**Figure 2:**
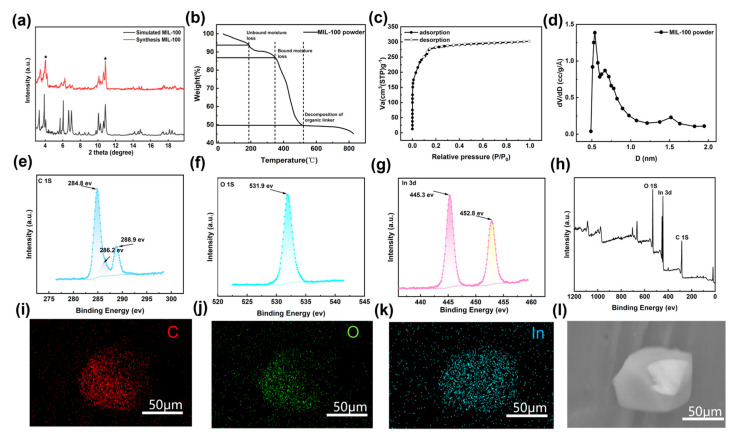
(**a**) XRD patterns for MIL-100(In) and simulated MIL-100(In); ★ represents the characteristic peaks for MIL-100(In); (**b**) TGA curve for MIL-100(In); (**c**) N_2_ adsorption and desorption isotherms for MIL-100(In) at 77 K; (**d**) pore size distribution of MIL-100(In) calculated from 77 K N_2_ adsorption isotherm based on the Horvath–Kawazoe model; (**e**–**h**) XPS spectra and (**i**–**l**) EDS spectra of MIL-100(In).

As shown in Figure 2a, the diffraction peaks of synthesized MIL-100(In) were consistent with the standard spectrum after multiple repeated washings, with the main characteristic diffraction peaks appearing near 4° and 11° [23], and their clear and sharp peak shapes indicated that the powders have a high degree of crystallinity [25,26,27]. Due to the strong alumina peaks, the MIL-100 characteristic peaks were unclear. Therefore, the subsequent XRD scan range involving the membrane was narrowed to 3~20° to better identify the peaks. The thermal decomposition temperature (Figure 2b) of MIL-100(In) was 343 °C, aligning with the previous work [28]. Specifically, the weight loss before 200 °C mainly originated from the removal of uncoordinated water from the pores. The weight loss before 343 °C was mainly due to the desorption of water molecules coordinated on the trimetallic oxygen cluster and carboxylic acid ligands that were not involved in the reaction. When the temperature was higher than 343 °C, the MIL-100(In) structure started to decompose irreversibly due to the disintegration of the ligand molecules, and the curve decreased sharply. Nitrogen (N_2_) adsorption at 77 K was measured to assess the micropore characteristics of MIL-100(In) (Figure 2c). The sample showed Type-I adsorption characteristics, which indicated that MIL-100(In) belonged to the microporous materials. In addition, based on the adsorption data, the Brunauer–Emmett–Teller (BET) surface area and micropore volume were determined by t-plot analysis with a surface area of 1264.0 m^2^/g and a pore volume of 0.48 cm^3^/g (Table 1); the pore size obtained by the Horvath–Kawazoe model was 0.77 nm (Figure 2d), which was consistent with prior work [29]. XPS was used to investigate the different elemental compositions on the surface of the sample (Figure 2e–h); MIL-100(In) consisted of three elements, C, O, and In. Among them, the In 3d XPS spectrum showed two characteristic peaks at 445.3 eV and 452.8 eV, which indicated the presence of In^3+^ during the synthesis of MOF. The C 1s spectrum showed three peaks of 284.8 eV, 288.9 eV, and 282.2 eV, where the peaks located at 284.8 eV and 288.9 eV corresponded to the electronic binding energies of phenyl and the carboxyl group [30,31], and the peak located at 286.2 eV were attributed to the carbon on the surface of the sample [32]. In addition, the elemental distribution of the samples was analyzed by SEM combined with EDS (Figure 2i–l). The sample surface was covered with a large amount of C, O, and In elements, which confirmed that MIL-100(In) consists of these three elements, which was in agreement with the previous XPS characterization results.

### 3.2. Characterization of MIL-100(In) Membrane

**Figure 3:**
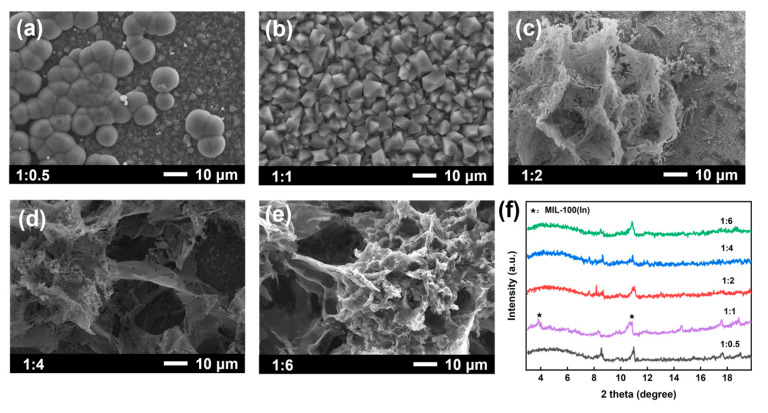
Surface SEM images of MIL-100(In) membranes prepared with different metal-to-ligand molar ratios: (**a**) 1:0.5; (**b**) 1:1; (**c**) 1:2; (**d**) 1:4; (**e**) 1:6; and (**f**) the corresponding XRD patterns.

The synthesis of MOF membranes is significantly influenced by key parameters, including the composition/concentration of the synthetic solution and the metal-to-ligand ratio, which critically affect membrane formation and performance [33]. Thus, the effect of different metal-to-ligand ratios on MIL-100(In) membranes was investigated. At a 1:0.5 ratio, the surface exhibited numerous spherical particles with a nonuniform distribution (Figure 3a). When the ratio was adjusted to 1:1, well-defined octahedral crystals formed, yielding a dense and continuous crystalline membrane (Figure 3b). Further increasing the ratio to 1:2~1:6 resulted in amorphous-like crystals and incomplete substrate coverage (Figure 3c–e). XRD analysis revealed that only the 1:1 ratio produced distinct MIL-100(In) characteristic peaks at 4°and 11°, while other ratios failed to show these peaks (Figure 3f). Based on these findings, the optimal metal-to-ligand ratio for fabricating high-quality MIL-100(In) membranes was determined to be 1:1.

**Figure 4:**
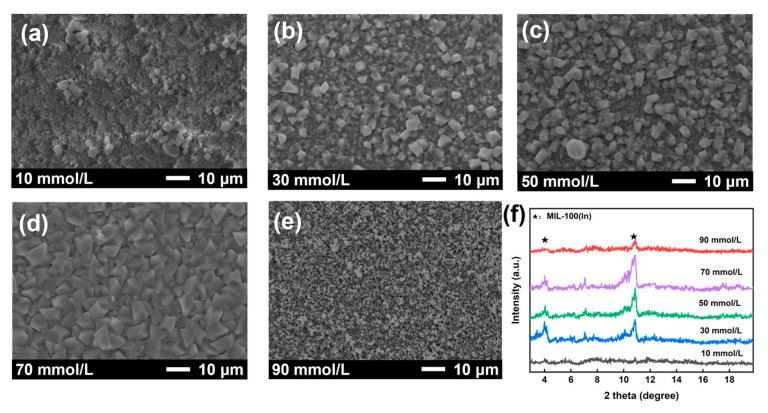
Surface SEM images of MIL-100(In) membranes prepared with varying precursor concentrations: (**a**) 10 mmol/L; (**b**) 30 mmol/L; (**c**) 50 mmol/L; (**d**) 70 mmol/L; (**e**) 90 mmol/L; and (**f**) the corresponding XRD patterns.

The rate of nucleation and growth of MOFs are significantly affected by the concentration of nutrients in the reaction solution [34]. Therefore, this concentration effect was investigated at a fixed 1:1 metal-to-ligand ratio. At concentrations lower than 30 mmol/L, no visible particles formed on the substrate (Figure 4a), and XRD showed no characteristic peaks at 4° and 11°, indicating insufficient nucleation due to low nutrient availability. When increasing the reactant concentration to 30~50 mmol/L, nucleation occurred but produced small (~1 μm), sparsely distributed particles that failed to form a continuous membrane (Figure 4b,c). Further increasing the concentration to 70 mmol/L yielded well-developed 5 μm particles and formed a dense and defect-free membrane (Figure 4d), suggesting optimal balance between nucleation and growth rates. This was confirmed by the appearance of distinct XRD characteristic peaks at 4° and 11° (Figure 4f). When further increasing to 90 mmol/L, only a sparse particle deposition was observed on the substrate (Figure 4e). This phenomenon can be attributed to the excessively high concentration of the reaction solution, which promoted rapid homogeneous nucleation in the bulk solution at the expense of heterogeneous nucleation on the substrate surface [35]. Under such conditions, the overwhelming majority of particle formations occurred in solution rather than on the substrate, resulting in poor surface coverage. In summary, 70 mmol/L was identified as the optimal concentration in membrane fabrication.

**Figure 5:**
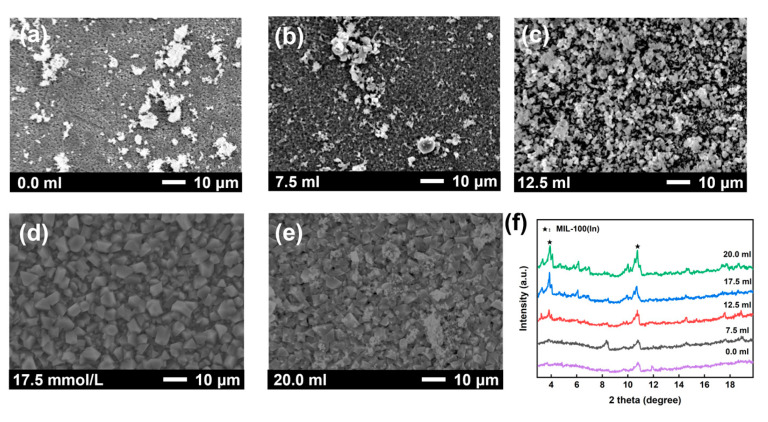
Surface SEM images of MIL-100(In) membranes prepared with different DMF amounts: (**a**) 0.0 mL; (**b**) 7.5 mL; (**c**) 12.5 mL; (**d**) 17.5 mL; (**e**) 20.0 mL; and (**f**) the corresponding XRD patterns.

The solvent plays a crucial role in MOF membrane fabrication. It has been shown that the morphology, structure, or properties of a MOF are closely related to the type and composition of solvents [36,37]. Especially in this work, DMF, as a common polar organic solvent, may lead to the easy deprotonation of H_3_BTC at atmospheric pressure [38]. Thus, systematic investigation of DMF amounts can reveal its critical impact on MIL-100(In) membrane formation. Without DMF, no MIL-100(In) particles were observed on the substrate due to low solubility (Figure 5a), and XRD also showed no characteristic peaks at 4°. As DMF usage increased, enhanced BTC solubility led to higher effective ligand concentration, promoting particle nucleation and growth (Figure 5b–e). Optimal results were achieved with 17.5 and 20 mL of DMF, producing a uniform, continuous membrane with a homogeneous particle size distribution and no visible defects. The corresponding XRD pattern (Figure 5f) exhibited well-defined MIL-100(In) diffraction peaks at 4° and 11°, confirming excellent crystallinity and structural integrity. This may be due to the fact that the solubility of BTC increases with the increase in DMF in the reaction medium, which promotes the coordination of carboxyl groups with indium ions and the formation of more stable structures [39]. Therefore, 17.5 mL of DMF was chosen as the optimal usage to obtain the MIL-100(In) membrane with fewer costs.

**Figure 6:**
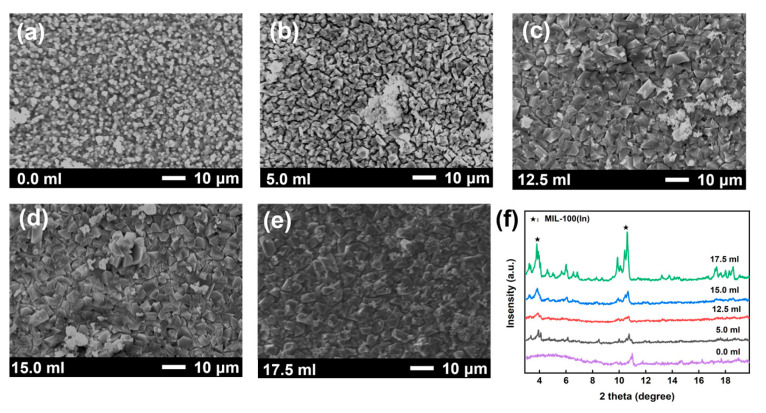
Surface SEM images of MIL-100(In) membranes prepared with different ethanol amounts: (**a**) 0.0 mL; (**b**) 5.0 mL; (**c**) 12.5 mL; (**d**) 15.0 mL; (**e**) 17.5 mL; and (**f**) the corresponding XRD patterns.

Previous studies have shown that in addition to the amount of solvent, their composition also has an effect on the shape and size of the MOF [40,41,42]. Ethanol, as a polar co-solvent, modifies reaction conditions by altering system pH and promoting ligand deprotonation, thus slowing down the nucleation rate and facilitating crystal growth [43,44,45,46,47,48]. Therefore, the key role of ethanol in the formation of MIL-100(In) membranes was investigated. As shown in Figure 6a, without ethanol, only amorphous particles formed on the substrate, with absent XRD peaks (4°) indicating failed crystallization. Increasing ethanol (5.0~15.0 mL) progressively enhanced crystallinity, evidenced by emerging characteristic peaks and particle growth (from 5 μm to 8 μm), demonstrating ethanol’s ability to modulate nucleation kinetics and extend growth periods. However, insufficient ethanol led to discontinuous films with surface cracks (Figure 6b–d). Optimal results emerged at 17.5 mL of ethanol (Figure 6e), as the nucleation and growth at this time tended to be stable, enabling the formation of a continuous and dense membrane. The findings established 17.5 mL of ethanol as the optimal amount for high-quality MIL-100(In) membranes.

**Figure 7:**
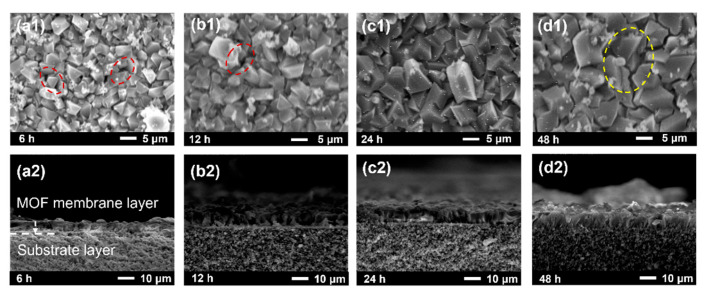
Surface (**a1**–**d1**) and cross-sectional (**a2**–**d2**) SEM images of MIL-100(In) membranes prepared at different synthesis times (6~48 h). (Pinholes are marked with red circles; cracks are marked with yellow circles).

Generally speaking, the distribution of particles and the crystallization process depend strongly on the reaction time, thus affecting membrane formation [49,50]. Hence, the effect of reaction time on the growth of MIL-100(In) membranes was investigated. With 6 h of reaction, an initial particle layer formed (Figure 7(a1,a2)), demonstrating that MIL-100(In) has rapid nucleation kinetics behavior. However, this early-stage membrane remained discontinuous and thin (about 5 μm) from the cross-section view. Extending the reaction time promoted significant crystal growth and improved particle intergrowth, evidenced by an increased particle size (from 5 μm to 8 μm) and denser packing (Figure 7(b1,b2)). An optimal membrane occurred at 24 h, where crystal growth was in the direction of the film layer, pinhole defects were minimized, and membrane integrity improved significantly (Figure 7(c1,c2)). As the reaction time continued to increase, cracks appeared on the surface of the membrane due to the continuous growth of crystals (Figure 7(d1,d2)). In summary, a continuous, dense membrane with uniform particle distribution can be obtained at 24 h.

**Figure 8:**
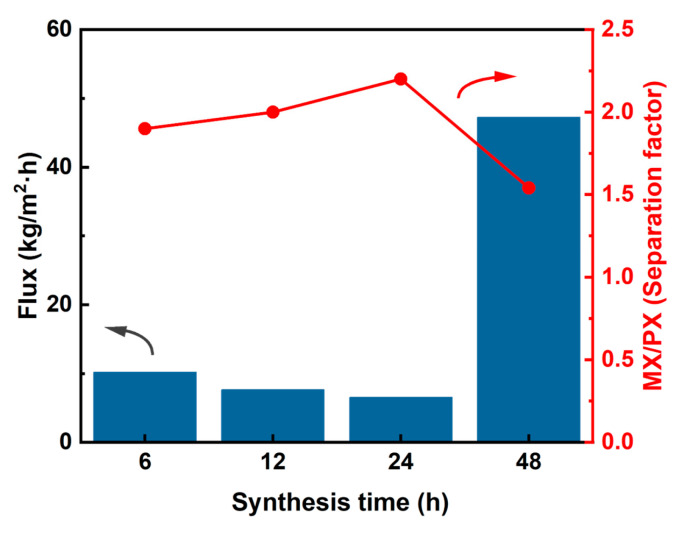
Pervaporation performance of xylene isomers of MIL-100(In) membranes prepared over different synthesis times (from 6 h to 48 h).

Pervaporation performance was evaluated to determine the optimal reaction time. As shown in Figure 8, extending the reaction time from 6 to 24 h resulted in a flux decline from 10.2 kg·m^−2^·h^−1^ to 6.5 kg·m^−2^·h^−1^, accompanied by an improved separation factor from 1.9 to 2.2. This inverse relationship between flux and the separation factor reflects the characteristic trade-off in MOF membranes [51,52], where the thicker the membrane layer is, the smaller its permeation flux is and the higher its separation factor is. The 24 h synthesized membrane exhibited balanced performance, maintaining an adequate flux of 6.5 kg·m^−2^·h^−1^ and achieving an optimal separation factor of 2.2. However, further extension to 48 h caused dramatic changes: flux surged to 47 kg·m^−2^·h^−1^ while the separation factor dropped to 1.54. This performance deterioration correlated with the membrane cracking observed in SEM images (Figure 7(d1,d2)), confirming structural failure with prolonged reaction times. Comprehensive analysis of both morphological characteristics (Figure 7) and separation performance (Figure 8) demonstrated that 24 h represented the optimal compromise between structural integrity and separation efficiency.

**Figure 9:**
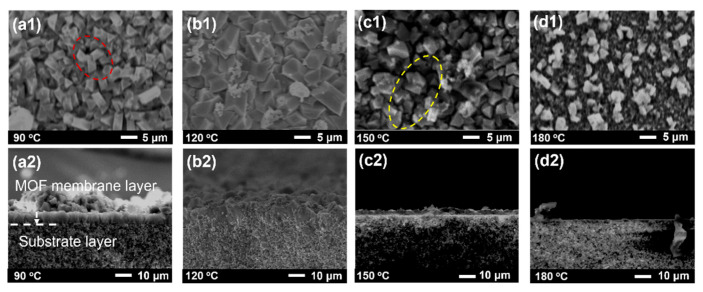
Surface (**a1**–**d1**) and cross-sectional (**a2**–**d2**) SEM images of MIL-100(In) membranes prepared at different synthesis temperatures (90~180 °C). (Pinholes are marked with red circles; cracks are marked with yellow circles).

The reaction temperature is a critical parameter influencing the growth kinetics and morphological characteristics of MOF crystals, as it governs both nucleation and particle growth processes. Elevated temperatures typically promote nucleation at the expense of crystal growth, often resulting in a higher density of smaller particles [53,54,55]. To elucidate these effects on MIL-100(In) membrane formation, the effect of different reaction temperatures on MIL-100 membranes was investigated. When the temperature was 90 °C, there was a layer of about 4 μm of MIL-100(In) particles on the surface, exhibiting poor intergrowth and failing to develop a continuous membrane (Figure 9(a1,a2)). Increasing the temperature to 120 °C significantly enhanced crystal growth, yielding larger particles with improved intergrowth that coalesced into a dense, defect-free continuous membrane (Figure 9(b1,b2)). However, further elevation to 150 °C induced explosive nucleation due to the rapid reaction rate, which resulted in the emergence of small, non-symbiotic particles (Figure 9(c1,c2)). Remarkably, at 180 °C, few particle deposition was observed, which indicated that the continuous increase in reaction temperature led to an accelerated particle nucleation rate instead of a particle growth rate (Figure 9(d1,d2)). These results demonstrated the delicate balance between nucleation and growth rates in determining MOF membrane quality. Finally, 120 °C was chosen as the optimal temperature for producing continuous MIL-100(In) membranes.

**Figure 10:**
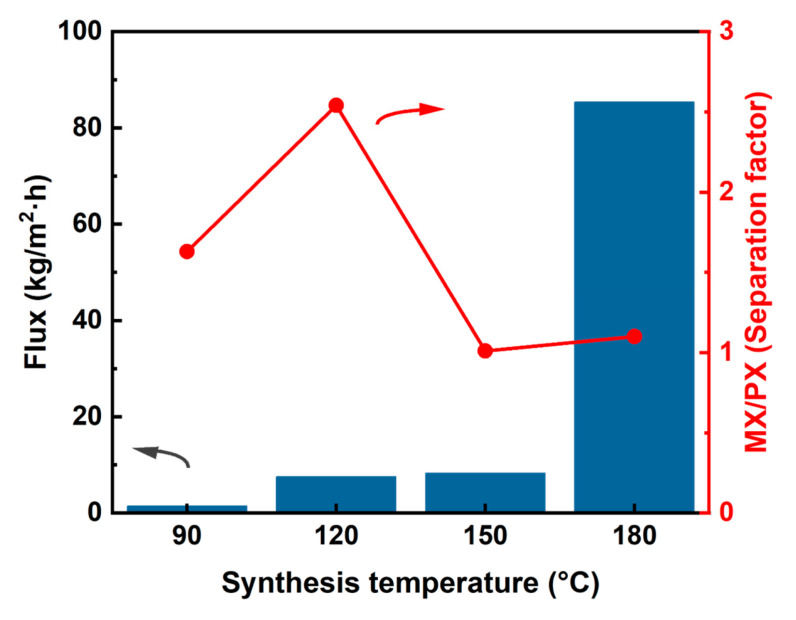
Pervaporation performance of xylene isomers of MIL-100(In) membranes prepared at different synthesis temperatures (from 90 °C to 180 °C).

The pervaporation performance of MIL-100(In) membranes was investigated under varying synthesis temperatures. As shown in Figure 10, the total flux increased from 1.4 kg·m^−2^·h^−1^ to 84 kg·m^−2^·h^−1^ as synthesis temperature increased from 90 °C to 180 °C, which was attributed to the fact that the membrane becomes thinner with increasing temperature (Figure 9). Notably, the membrane achieved an optimal separation factor of 2.54 at 120 °C. This temperature represented a critical balance between membrane formation and performance. At 180 °C, excessive nucleation prevented continuous membrane formation, leading to drastically increased flux and the complete loss of separation capability, which was in line with the conclusions obtained from the SEM images (Figure 9(d1,d2)). These results demonstrated that 120 °C was the optimal synthesis temperature, producing MIL-100(In) membranes with both sufficient structural integrity and effective separation performance for xylene isomers. To sum up, when the metal-to-ligand ratio was 1:1, the concentration of the synthetic solution was 70 mmol/L, the volume ratio of the solvent was 3.5:3.5:1, the synthesis time was 24 h, and the synthesis temperature was 120 °C, a dense MIL-100(In) membrane without defects can be obtained. Under these optimized conditions, the synthesized MIL-100(In) membrane exhibited ideal morphological characteristics: it had a uniform crystal morphology, which is highly consistent with that reported in the literature [26,27]; the crystals are densely packed on the substrate; and adjacent crystals form a dense continuous membrane layer through co-growth, with no obvious intergranular defects (Figure 9(b1,b2)).

### 3.3. Separation Performance

#### 3.3.1. Temperature Effect and Long-Term Stability

**Figure 11:**
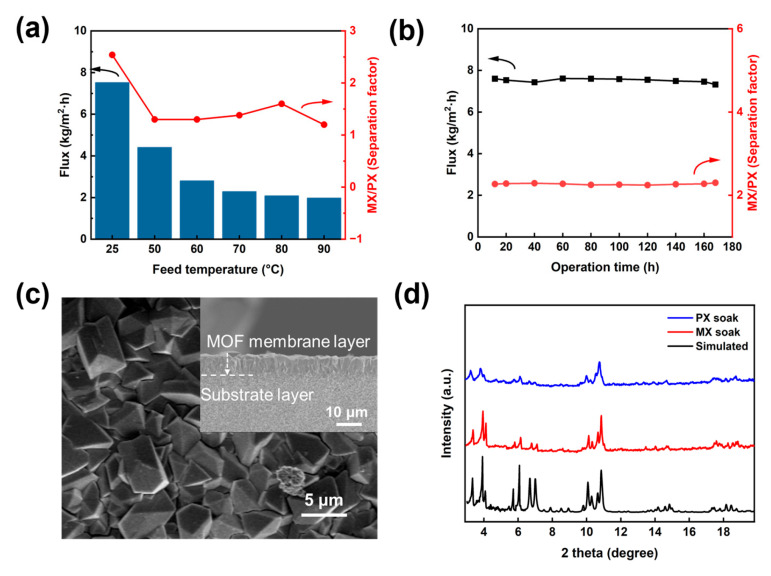
(**a**) Effect of feed temperature (25~90 °C) on separation performance. (**b**) Long-term stability test of the MIL-100(In) membrane for MX/PX (1:1) separation at 25 °C. (**c**) Surface and cross-sectional SEM images of the MIL-100(In) membrane after the measurement of xylenes separation. (**d**) XRD patterns of MIL-100 powders after the PX and MX soak.

Temperature plays a crucial role in membrane separation processes, significantly influencing molecular diffusion rates, adsorption behavior, and membrane pore structure stability. While elevated temperatures generally enhance molecular diffusion and permeation flux, they may simultaneously induce structural modifications in the membrane that compromise the separation factor [56,57]. Here, the effect of MIL-100(In) membranes on the separation of MX and PX mixtures at different feed temperatures was investigated. As shown in Figure 11a, optimal separation performance was observed at 25 °C, and the total flux showed a clear trend of decrease (form 7.6 kg·m^−2^·h^−1^ to 1.9 kg·m^−2^·h^−1^) as the feed temperature increased from 25 °C to 90 °C. Elevated temperatures serve to improve molecular mobility [15]. Due to its high degree of size compatibility with the MIL-100(In) pores, MX exhibited a preferential tendency to enter the framework. Upon interaction with the framework, MX molecules firmly adhered to the surface, progressively colonizing the available effective channel space and thereby inducing a diffusion impediment. Consequently, other molecules faced significant resistance, restricted by both intrinsic diffusional constraints and steric hindrance, which severely impeded their penetration. Furthermore, these strong interactions established a dynamic adsorption barrier that restricted the diffusive transport of MX itself [7,58,59]. These resistances resulted in the reduced flux and separation factor. These findings demonstrated that 25 °C represented the optimal operating temperature for achieving both high flux and effective xylene isomer separation with MIL-100(In) membranes.

Figure 11b showed the long-term pervaporation performance of the MIL-100(In) membrane during continuous operation at 25 °C for 168 h. Throughout the entire test duration, the membrane demonstrated excellent stability, with its permeation flux consistently maintained within the range of 7.5 ± 0.1 kg·m^−2^·h^−1^ and the separation factor stable at 2.5 ± 0.1, indicating that the MIL-100(In) membrane possessed outstanding long-term operational stability. After prolonged operation, no significant changes were observed on the surface or cross-section of the membrane (Figure 11c); additionally, after the same duration of immersion, the XRD patterns of the powders also showed no significant changes (Figure 11d), further confirming the stability of the MIL-100(In) membrane in the separation of xylene isomers.

Membrane separation technology shows great potential for application in the separation of xylene isomers. To systematically evaluate the separation efficiency of the MIL-100(In) membrane, this study reviewed the performance parameters of various membrane materials reported in the literature (including polymer membranes, mixed-matrix membranes, and MOF membranes) and compared them with the MIL-100(In) membrane prepared in this work. As shown in Table 2, although the MIL-100(In) membrane exhibited relatively low separation selectivity for xylene isomers, its flux (7.6 kg·m^−2^·h^−1^) was significantly higher than that of traditional membrane materials [60]. Notably, during a 168-h stability test, the performance degradation rate of the MIL-100(In) membrane was less than 5%, demonstrating excellent stability. This unique combination of high flux and high stability makes the MIL-100(In) membrane highly promising for industrial-scale xylene isomer separation applications. Moreover, this study employed a green and efficient in situ solvothermal synthesis technique to prepare MIL-100(In) membranes, offering significant advantages in terms of material cost and environmental friendliness, thereby laying a solid foundation for its industrial application. In the future, membrane surface modification may further enhance its separation selectivity to meet the stringent requirements of industrial production.

#### 3.3.2. Separation Mechanism

**Figure 12:**
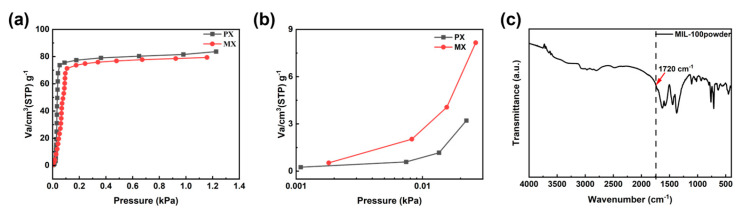
Single-component vapor adsorption isotherms of xylene isomers on MIL-100(In) at (**a**) 298 K and 0~1.4 kPa, (**b**) 298 K and 0~0.03 kPa, and (**c**) FTIR spectra of MIL-100(In) powders.

The adsorption and separation performance of MIL-100(In) for paraxylene isomers (PX and MX) was investigated using a single-component vapor adsorption test. The experimental results showed that adsorption reached saturation at 0.1 kPa, with adsorption capacities of 83.704 cm^3^(STP)/g and 79.398 cm^3^(STP)/g for PX and MX, respectively (Figure 12a). However, there were significant differences in adsorption behavior in the low-pressure range (<0.03 kPa): the adsorption isotherm of MX exhibited a steeper upward trend (Figure 12b), with a higher initial adsorption slope than PX, indicating stronger intermolecular forces between MX and MIL-100(In), including π-π stacking interactions and interactions with multiple oxygen sites within the framework. Other work reported similar behavior. Xing et al. found that the adsorption isotherm of MIL-160 for MX is steeper in the low-pressure region, indicating that it has a higher affinity for MX [22,65,66]. This differentiated low-pressure adsorption behavior resulted in MX having a significantly higher adsorption affinity than PX.

PX molecules (0.67 nm) adopt a linear, symmetric configuration that requires significant tilting to fit within MIL-100’s pores (0.77 nm), resulting in poor spatial matching and weak interactions. In contrast, the MX molecule (0.71 nm) exhibits a curved asymmetric structure, forming a more compact morphology, which may better accommodate the pore geometry, and the molecule is able to form a symmetric stacking structure when maximizing the use of pore space within the channel, so that the two methyl groups of MX can bind to the binding sites of the pore channel synergistically, thus exhibiting a strong affinity. This phenomenon aligns with the “proportional stacking” mechanism proposed by Torres-Knoop et al. [67,68], where xylene molecules achieve efficient, ordered packing in MOF pores. In addition, due to the highly similar physicochemical properties of xylene isomers, it is difficult to achieve effective molecular recognition by relying only on a single binding site. Therefore, MOF materials featuring multiple adsorption sites can overcome this limitation by establishing cooperative interactions through their densely distributed negative oxygen sites, thus exhibiting excellent selective recognition of isomers [22]. MIL-100(In) is particularly effective in this regard due to its oxygen-rich organic ligands and multiple binding sites within the framework structure. Furthermore, from the IR spectral analysis, a strong absorption band at 1720 cm^−1^ was observed (Figure 12c), and this characteristic peak was attributed to the stretching vibration of the C=O bond in the carboxyl group (ν(C=O)), which may be due to the strong interaction between the carboxyl oxygen atom in the trimesic acid molecule and the metal atoms (In) in the M_3_O trimer through the CO-M single-point coordination mode [26,69,70], which resulted in a C=O bond shift that occurs and exhibits a clear closing peak at 1720 cm^−1^. In addition, the trimesic acid molecule acted as one of the terminal molecules of the M_3_O trimer, suggesting that the pore surface of MIL-100(In) was endowed with a polar character due to the carboxylic acid fraction [71]. Therefore, this multi-site material preferentially exhibits the preferential adsorption of MX with higher polarity.

Moreover, as demonstrated in numerous studies [72,73,74,75,76], host–guest interactions represent another common mechanism for xylene separation in MOFs. In the case of MIL-100(In), the aromatic ring of the guest molecule MX and the host metal–organic skeleton with aromatic ligands undergo π-π stacking interactions, which further enhances the strong adsorption of MX. These combined effects—optimal molecular packing, synergistic binding, and enhanced π-π interactions—contribute to the membrane’s superior MX separation factor and permeation performance.

## 4. Conclusions

The separation of xylene isomers remains a significant industrial challenge due to their nearly identical physicochemical properties. Herein, a high-performance MIL-100(In) membrane for MX/PX separation was synthesized on ceramic supports via in situ solvothermal methods. The membrane’s exceptional separation factor originates from the optimal match between the kinetic diameter of MX (0.71 nm) and the pore size of MIL-100 (0.77 nm), coupled with preferentially interacting with more polar MX. The membranes demonstrated outstanding pervaporation performance, achieving a high flux of 7.6 kg·m^−2^·h^−1^ and a separation factor of 2.54 for equimolar MX/PX feed mixtures at room temperature. This work not only represents a significant advancement in xylene isomer separation technology but also highlights the potential of membrane separation technology for practical industrial applications. The successful fabrication of high-performance MIL-100(In) membranes on ceramic supports provides a promising platform for scale-up production and implementation in industrial separation processes.

## Figures and Tables

**Table 1:** Pore volume and specific surface area of MIL-100 powder.

Material	Bet Surface Area	Total Pore Volume	Average Pore Diameter
MIL-100(In)	1264.0 m^2^/g	0.48 cm^3^/g	0.77 nm

**Table 2:** Performance comparison of various membranes for the separation of xylene isomers.

Material	Feed Composition	Separation Factor	Flux (g·m^−2^·h^−1^)	Ref.
Mixed-matrix membranes for the separation of xylene isomers				
PGS/PAM	PX/MX (10/90)	1.64	47.3	[9]
PGS/PAM	MX/PX (10/90)	1.4	37.3	[10]
SPMMM-Fu	PX/MX (10/90)	39.73	34.6	[11]
ꞵ-CD/PVA	PX/MX (10/90)	2.96	95.0	[12]
silicalite-1/PAAS	PX/MX (15/85)	2.68	342.8	[13]
ꞵ-CD-EGDE/PVA	PX/MX (10/90)	1.34	58.0	[14]
Other membranes for the separation of xylene isomers				
MFI	PX/MX (50/50)	1.00	200.0	[6]
CA-DNP	PX/MX (50/50)	1.30	7.3	[7]
PE	PX/MX (50/50)	1.2	0.5 *	[8]
Metal–organic framework membranes for the separation of xylene isomers				
UiO-66	PX/MX (50/50)	1.20	370.0	[16]
MIL-160	PX/MX (50/50)	40.50	486.0	[15]
MOF-5	PX/OX (50/50)	1.24	162.5 ^#^	[61]
MOF-5	PX/OX (50/50)	2.22	169.5	[62]
	PX/MX (50/50)	1.95	177.0	
ZIF-68	/	/	492.0	[63]
UiO-66	PX/OX (50/50)	4.83	2.8 **	[64]
Zn_2_(BDC)_2_DABCO	MX/PX (50/50)	1.62	2627.0	[58]
MIL-100(In)	MX/PX (50/50)	2.54	7600.0	This work

* Unit is L·m^−2^·h^−1^·bar^−1^; ^#^ Unit is 10^−5^ mol/m^2^·s; ** Unit is 10^−8^ mol·m^−2^·s^−1^·Pa^−1^; Feed composition (A/B, A represents the easily permeable component).
